# Effectiveness of Different Teaching Resources for Forming the Concept of Magnitude in Older Preschoolers with Varied Levels of Executive Functions

**DOI:** 10.11621/pir.2022.0405

**Published:** 2022-12-30

**Authors:** Aleksander N. Veraksa, Anastasia N. Sidneva, Margarita S. Aslanova, Valeria A. Plotnikova

**Affiliations:** a Lomonosov Moscow State University, Moscow, Russia

**Keywords:** Teaching resources, regulatory functions, executive functions, symbol, elementary mathematical conceptions, older preschool age

## Abstract

**Background:**

Studies have shown the great importance of early mathematical development as a predictor of subsequent success, which poses the question of how to organize preschool mathematical education with a view to the children’s age characteristics, including their cognitive development. In other words, mathematical concepts and actions should be formed with the help of teaching resources appropriate to the child’s development.

**Objective:**

To determine the effectiveness of three teaching resources (examples, models, and symbols) in formation of the concept of magnitude in older preschoolers (ages 6–7) with different levels of executive function.

**Design:**

Four training programs (with 15 twenty-minute lessons each) were developed and conducted in a formative experiment for older preschoolers with different levels of development of executive functions. The lessons addressed the concept of magnitude (length, area, volume), using different types of teaching resources: exemplars (in traditional and game variants), models, and symbols. The total sample of 116 subjects (44% boys) was divided into 4 groups for each of the programs, plus a control group in which no sessions were conducted. The groups were equalized according to the initial level of development of concepts of magnitude and the level of development of executive functions.

**Results:**

There was a statistically significant increase in the quality of mastery of the concept of magnitude in three experimental groups (“symbolic,” “traditional,” and “traditional with imaginary characters”) compared with the control group. The formative effect of the “model-building” program showed no significant differences from the effect of the child’s natural development (the control group). We also showed that children with a low level of regulation learned mathematical concepts more effectively with the “symbolic” program; children with a medium level of regulation with the “symbolic” and any variant of the “traditional” program; and children with a high level of regulation with the “symbolic” and “model-building” programs.

**Conclusion:**

The findings underline the importance of both the type of teaching resources used and the level of development of voluntary regulation, when teaching mathematics to preschoolers.

## Introduction

Research in psychological and pedagogical science has demonstrated a significant influence of early mathematical skills on subsequent academic and social success, both in school and in adulthood (Jordan et al., 2009; Tikhomirova, 2021; Watts et al., 2018). The great importance of preschool mathematics teaching for subsequent development (Ritchie & Bates, 2013; Watts et al., 2014) indicates the need for organization of effective education at this age based on the specifics of child development in the preschool period. This is extremely important given the nature of modern preschool childhood: increasing demands on children’s intellectual development, and the discrepancy between children’s age requirements and characteristics and some models of education, leisure activities, and children’s products (Smirnova, 2019).

Following the cultural-historical and activity approaches, studies have shown (Aleksandrova, 2013; Davydov et al., 1996; Davydov & El’konin, 1966; Obukhova, 1972; Shinelis & Sidneva, 2020) that it is advisable to start introducing children to mathematical reality by mastering an elementary mathematical concept such as magnitude. Magnitude itself is usually determined using three comparison relations (a = b, a > b, a < b); examples of magnitudes that preschoolers constantly confront include length, area, volume, and quantity. The concept of magnitude is essentially a system-forming concept that underlies the concepts of number, function, and figure, and, accordingly, links three domains of mathematics: arithmetic, algebra, and geometry (Davydov, 1962).

According to P.Ia. Galperin’s theory of planned stage-by-stage formation of mental actions and concepts, any concepts new to the child should be learned as reference points for relevant actions, revealing the cultural and historical conditions for the origin of these concepts (Gal’perin, 1975). In studies conducted according to the theory of developmental learning and the theory of planned stage-by-stage formation of mental actions and concepts, it has been shown that from the psychological and logical/subject-related standpoints, the most complete and appropriate idea of magnitude is formed when learning actions of comparison (establishing the correspondence or non-correspondence of magnitudes) and measuring quantities using a conditional measure (how many times the measuring instrument fits into the given magnitude) to establish relationships between them (Davydov, 1962; El’konin, 1963; Frolova, 1963; Gal’perin & Georgiev, 1960). The use of a conditional measure makes it possible, first, to compare objects that cannot be directly placed upon each other, and second, to concretize the relationship between magnitudes and understand how much one magnitude is larger or smaller than another. When teaching is organized this way, the concept of “magnitude” is mastered as a necessary reference-point for a specific object-oriented action — the action of comparison and measurement (Gal’perin, 1976) — which allows us to say whether children have understood its essential features. However, even when the children perform the same actions, the organization of specific cognitive situations and tasks can be provided by various teaching methods. This raises the issue of the effectiveness of such methods.

### Teaching Resources and Age Characteristics of Preschoolers

Teaching resources are defined as anything that facilitates the transfer of knowledge in the instructional process (Salmina, 1988). This may include materials and study aids used in the classroom, the teacher’s narrative, etc. They may differ in form and content. However, the key point in achieving developmental effects is the difference among teaching resources according to their function in children’s action (Salmina, 1988). From this point of view, it is essential to consider teaching resources that:

1. Establish a meaningful purpose for the child’s action;
2. Provide a way for that action to be performed.

In this context, the term “resource” is used here in the sense of an instructional approach. However, the teaching resources used in the functions mentioned above may also be considered as psychological methods that allow one to master new types of activity (Vygotsky, 2004). Mastering the cultural system underlying one’s own cognitive activity is an important aspect of the cognitive development of a preschool child (El’konin, 1989; Karabanova, 2005; Venger, 1986). Accordingly, the child’s success in mastering mathematical content will depend on the appropriateness of the instructional approaches that are selected.

These instructional approaches and resources must first of all be consistent with the logic of amplification (Zaporozhets, 1986). In other words, the tools should “grow out of ” children’s natural activities, in which zones of proximal development (ZPD) are also created. Based on the features of children’s activities described in various studies (El’konin, 1978; Sarama & Clements, 2009; Shapovalenko, 2004; Shiiaan et al., 2021; Solovieva et al., 2021; Venger & Kholmovskaia, 1978), we have identified three possible types of teaching resources for this age group:

1. Exemplars (instructions or rules that are accepted by general agreement of the players), most actively used in games that have rules;
2. Models (diagrams, maps, plans, and other objects that allow the child to display the essential relationships between objects) encountered in children’s productive activities (model-building, construction, drawing, etc.);
3. Symbols (a magic wand, an imaginary letter, etc., in which the child singles out and maintains significant relationships through an emotional attitude to the situation being created), which are an essential part of the content of plot role-play.

In order to test the effectiveness of each type of teaching resource, we developed four different approaches to designing a curriculum to teach the concept of magnitude to older preschoolers:

1. Traditional approach with imaginary characters (the key teaching tool here is the exemplars introduced through imaginary characters);
2. Traditional approach (the key teaching tool is exemplars);
3. Model-building approach (the key teaching tool is models);
4. Symbolic approach (the key teaching tool is the symbol);

Although the teaching resources identified here have analogues in the free activity of most preschoolers, we believe that such tools can play different roles, depending on the particular characteristics of the child’s development. Voluntary self-regulation plays a key role in this development. Thus, for example, orientation to exemplars and rules appears significantly later than symbolization and model-building (these are the essence of mastering the substitutive function of game objects); such an orientation, closely related to voluntary regulation, appears only at the stage of already rather developed plot role-play (El’konin, 1978; Veraksa & Veraksa, 2016). Therefore, it seems to us fundamentally important to consider the effectiveness of various means of forming mathematical representations in the context of the development of voluntary self-regulation in preschoolers.

### Mathematical Development and Voluntary Self-Regulation at Preschool Age

When we speak of self-regulation in this paper, we rely on the concept of regulatory or executive functions (EFs) as developed by A. Miyake and colleagues (Miyake, 2000). According to this concept, EFs are a group of cognitive processes that provide for purposeful problem solving and adaptive behavior in new situations (Diamond, 2012); that is, they are metacognitive capabilities (Morosanova et al., 2021). Executive functions comprise three components: 1) working memory (visual and verbal); 2) cognitive flexibility (focusing attention and/or switching attention under changing conditions); 3) inhibitory control (the ability to suppress an impulsive reaction).

Research shows that EFs predict the future performance of preschoolers (Duncan et al., 2007) and are correlated with mathematical ability (Bull & Scerif, 2001; Clements et al., 2016; Jarvis, 2003). Thus, for example, inhibitory control and cognitive flexibility in preschool children are predictors of mathematical ability at an older age (Best et al., 2011; Espy et al., 2004). A low level of EF is associated with difficulties in mastering mathematical concepts (Ribner, 2020; Swanson, 2001). These results raise the question of the particular ways that mathematical education should be organized for children with different levels of development of executive functions.

### Research Hypotheses

We have suggested that symbolic representations are most appropriate in the preschool period, as these are more natural from the standpoint of preschoolers’ play activity, and contribute to self-expression (Veraksa & Veraksa, 2016). Techniques using exemplars are more specific to traditional schools, where the teacher serves as a conveyor of cultural models and most often passes them on to students in a directive form. Modeling tools may be difficult to master in that situation. On this basis, we advanced the following empirical hypotheses:

1. Children with a low level of development of EFs will learn mathematical concepts and skills more successfully when using symbolic representations. This hypothesis is based on the fact that symbolization at an early age greatly facilitates perception of the conditions of tasks (Veraksa et al., 2014; Veraksa et al., 2020) and will be the most effective means of their formation due to the symbolic nature of mathematical representations (Salmina, 1988).
2. Preschoolers with a high level of EF development will show the best results from the model-building approach. This is because visual models are more conducive to the cognitive development of children with a pronounced cognitive orientation (Venger, 1995).

## Methods

### Participants

The sample comprised 150 children aged 6–7 (mean age 6.9) attending kindergarten preparatory groups, of whom 65 were boys (43%) and 85 were girls (57%). The study was conducted in the 2021–2022 academic year. All participants attended a Moscow educational complex where the program “From Birth to School” was used as the base program. The study was approved by the Ethics Committee of the Faculty of Psychology of Lomonosov Moscow State University.

### Questionnaires

#### Methods for Mastering the Concept of Magnitude

A diagnostic toolkit was developed for use with preschool children to assess the quality and stability of their formation of elementary mathematical representations of magnitudes and their relationships.

This diagnostic technique included four types of tasks for each magnitude: length, area, volume. The children were given two tasks to solve for each magnitude, about the ability to compare objects; they were asked to select an object the same size as another one — for example, to find the rectangle with the same length as that shown in a drawing. Two tasks each for the ability to use a measuring instrument correctly (“who measured correctly?”): to apply it so that there is no empty space between measurements, to use equal measuring instruments, etc. Two tasks each for actually measuring a magnitude with a conditional measuring instrument and recording the result with labels or a number (“how many times does the measuring instrument fit in this magnitude?”). Two tasks each to understand how the number depends on the measuring instrument used (the larger the measuring instrument, the fewer times it fits into the magnitude). Two more assignments were included for making sets (“what would be left over if such and such sets were used?”). The ability to put together complete sets was not specifically targeted in this study during the lessons, so we considered these tasks to be within the children’s zone of proximal development (ZPD). The tasks for making sets were assessed depending on the amount of assistance provided to the child by the tester and the correctness of the answer: the preschooler received 2 points for correctly solving the task independently, 1 point for solving the task correctly with the help of the tester’s prompts; 0 points if the task is not solved or is solved incorrectly even with the help of an adult. The formation of concepts of length and area was assessed from 0 to 10 points; of volume from 0 to 7 points; and for tasks in the ZPD from 0 to 4. The total possible score was 31 points. Diagnosis of mathematical concepts and skills was performed individually with each child.

#### Methods for Assessing the Development of Executive Functions

Recent studies show that the level of executive functions is significantly associated with children’s success in mastering mathematical content (Clements et al., 2016; Veraksa et al., 2020); therefore, we used the level of development of executive functions as the criterion for dividing the children into groups. To measure students’ EF, we used the NEPSY-II subtests (Korkman et al., 2007) for visual memory (Memory for Designs) and auditory working memory (Sentence Repetition), inhibition and switching (Naming and Inhibition, Statue), and also cognitive flexibility (The Dimensional Change Card Sort) and visual-spatial memory (Schematization). This allowed us to measure various components of preschoolers’ cognitive processes. The diagnosis of EFs was performed with children on an individual basis during two meetings with each child.

The NEPSY-II Memory for Designs subtest was used to assess working visual memory. This test includes the following final scores: content scores are awarded for correctly remembering picture details (maximum 46 points); spatial scores reflect how correctly the child remembers the configuration of a picture (maximum 24 points); and bonus scores are awarded to the child for correctly remembering and looking at both dimensions simultaneously (maximum 46 points). The three indicators are summed up in the final score (maximum 116 points).

Verbal working memory was assessed using the NEPSY-II “Sentence Repetition” subtest, which consists of 17 sentences that gradually become more difficult to remember due to their length and grammatical structure. Children receive 2 points for each sentence they repeat correctly; one point if they make one or two mistakes in the repetition by skipping, replacing or adding words, or changing the order of words; and if the child makes three or more mistakes or does not answer, no points are awarded. The assignment is terminated if the child receives 0 points four times in a row.

The NEPSY-II “Naming and Inhibition” subtest assesses information-processing speed and inhibition of impulsive reactions. It consists of two blocks: a series of white and black circles and squares and a series of white and black arrows showing different directions (up and down). Two tasks were performed with each series of pictures: a task to identify the form (in this case, the child simply had to quickly name the forms that he saw) and an inhibition task. In the latter case, the child had to do everything contrariwise: for example, if he saw a square, he was supposed to say “circle” and so on. For each task, the researchers recorded the number of mistakes the child made and corrected or could not correct, as well as the time it took to complete the task.

“The Dimensional Change Card Sort” test (Zelazo, 2006) was used to assess cognitive flexibility. This technique consists of three tasks for sorting cards. First, the children must arrange the cards by color, shape, and then follow a complex rule: if the card has a frame, they must sort it by color, and if there is no frame, they must sort it by shape. For each correctly sorted card, the child receives 1 point; at the end, the number of points for each series is calculated (maximum 6, 6, and 12 points, respectively), and then the total score for all tasks is calculated (maximum 24 points).

We used the “Statue” subtest (NEPSY-II, Korkman et al., 2007) to assess “hot” self-regulation and physical inhibitory control. In this test, the child is instructed to remain motionless with eyes closed for 75 seconds, inhibiting impulsive reactions to distracting sounds. An assessment is performed for each 5-second interval: 2 points are awarded if the child did not make any mistakes in the 5-second interval, 1 point is given if 1 mistake was made, and 0 points if 2 or more mistakes were made. Large movements of the arms, body, legs, head, opening of the eyes, vocalization or laughter are all considered errors. The total score (max. 30) and the number of errors are calculated for three categories: movements, eyes, and sounds.

We used the “Schematization” technique to assess planning and checking and visual-spatial orientation. Here, children are asked to find a “route” through an extensive system of streets, using the notation of this route with the help of a diagram and/ or a conditioned image in the form of a system of landmarks. The child has to take into account the sequence of landmarks and/or the direction of turns.

#### Methods for Assessing Intellectual Development

As a supplement, non-verbal intelligence was diagnosed using Color Progressive Matrices by D. Raven (Raven & Kort, 1997). In this technique, the children need to choose one of the six proposed images in order to complete a drawing while following a certain logic. The technique contains three series of 12 tasks (maximum score — 36).

All the techniques were presented to the children in digitized form with a mobile app.

### Procedure

The study comprised several stages. First, the cognitive processes of children were assessed using the NEPSY-II subtests “Card Sorting” and “Schematization,” as well as D. Raven’s matrices. After evaluating their EF, the children were divided into three subgroups by level of cognitive development (low, medium, high) according to the results of cluster analysis (K-means clustering) performed in IBM SPSS Statistics 22. Before the beginning of the experimental sessions, a pre-test of mathematical concepts and skills was conducted using the authors’ diagnostic tools.

Next, participants from each subgroup with low, medium, and high EF levels were randomly assigned to four experimental groups and one control group, so that the ratio of participants in the groups was uniform. For each approach, 15 experimental sessions lasting 15–20 minutes were held in mini-groups of 3–4 children. The sessions occurred twice a week in the first half of the day in the groups at the kindergarten. The control group did not attend any special sessions.

The sessions were completed for all approaches simultaneously, after which a post-test of mathematical conceptions and skills was performed, similar to the baseline diagnostics in the experimental and control groups. A month after the experimental sessions, some of the children took part in a delayed post-test.

### Formative Sessions

The programs we developed for the formation of concepts of magnitudes corresponded to the types of methods that “grow out of ” the natural activity of preschool children.

In the first program (“traditional”), an exemplar was offered as the main instruction for performing actions. The children were given specific instructions on how to perform an action as a way of solving a problem (for example, “here is how you measure with a ruler,” “look how I do it,” etc.). The meaningfulness of the tasks was not specifically addressed; the children were presented with tasks such as “measure,” “compare,” “find one that’s the same.” Why this had to be done was not discussed. Note that analysis of modern Russian programs and mobile apps for preschoolers has shown that the main teaching resource used is the exemplar, a rule taught to the children for performing a task or action by demonstration of the action, while the need to use the mathematical concepts and actions is given as an external condition (Aslanova et al., 2020; Sidneva et al., 2021).

In the second program (“traditional with imaginary characters”), the concept of magnitude was introduced in exactly the same way as in the first program: through a directive instruction about the mode of action and tasks that did not disclose the meaning of this action. However, the tasks given to the children were presented by characters in a game (Dumbo, Wizard, etc.), depicted in a colorful picture. These characters did not perform a symbolic function, nor did they help to make the task more meaningful. They were an external game element, introduced in order to evaluate the role of this type of game element, while maintaining the basic orientation to the exemplar.

In the third program (“model-building”), we introduced the concept of magnitude and worked on it through design tasks (“choose a suitable column for the building,” “what kind of tiles can be used to lay the floor in the bathroom?”). Here, meaningfulness was determined by, on the one hand, a real everyday or engineering situation that needs to be resolved, and on the other, the child’s desire to act like an adult, for example, like an engineer or like Dad, who repairs things. The solution was introduced as something that could help solve this type of problem. When actions with real objects were difficult (for example, they are too heavy, big, or fragile, or you need to perform the action right the first time so as not to have to redo the repair, etc.), the problem could be solved by constructing a model of the real objects (a diagram, drawing, or other type of model) and using it to test hypotheses. Using various models and schematized methods, the children were able to learn generalized information about the essential properties of the real world (Shapovalenko, 2004). And research has shown that it is indeed through manipulation of such models, that both the development of initial mathematical concepts and the formation of the main mental neoformations take place (Venger, 1978).

In the fourth program (“symbolic”), the concept of magnitude was introduced and worked out through tasks of helping an imaginary character (for example, “pour the same amount of the water of life to save the queen,” “help Winnie the Pooh find his way home from the dark forest”) that created emotional meaningfulness for the goal of the action. The means of solving the problem situation were also symbolic objects (for example, a magic ball for measuring a route; umbrellas that can protect a drawing on asphalt from rain; a magic cup). These symbolic representations established the problem situation, key points of orientation, and relationships for its solution, becoming reference points for mastering the concept of magnitude. In this case, the children did not need a model of the action; they themselves could construct the necessary action based on the symbolic image of the situation, since the symbol as a cognitive tool facilitates perception of the conditions of the task in a situation of uncertainty (Veraksa et al., 2014; Veraksa et al., 2020 ). And at the same time, it ensures the children’s emotional involvement in the activity (Leont’ev, 2000; Veraksa et al., 2015)

The programs we developed are identical in terms of the object-specific actions performed by the children: measurement and selection with the help of conditional measures, but they differed in the teaching resources used. The following concepts were chosen as formed concepts in all the programs: length (including width and height), area, and volume.

Experimental sessions were conducted by specially trained teachers, who did not themselves perform the preliminary and subsequent testing.

## Results

The final sample of the formative experiment included 116 preschoolers who had gone through both EF diagnostics and a pre-test in mathematics. The children who were included in the formative experiment did not differ from those excluded from it in the development of their visual working memory (Chi-square = 0.9, *p* = 0.3), inhibition and switching (Chi-square = 0.3, *p* = 0.6), cognitive flexibility (Chi-square = 1.9, *p* = 0.16), visuospatial memory (Chi-square = 0.01, *p* = 0.9), and verbal intelligence (Chi-square = 0.2, *p* = 0.6). However, differences were found in their auditory-verbal working memory (Chi-square = 5.7, *p* = 0.01), which we did not consider significant, since research has shown that auditory-verbal working memory is weakly associated with mathematical development (Bull & Johnston, 1997)

The participants comprised 51 boys (44%) and 65 girls (56%). No significant statistical differences were found in the level of mathematical abilities for boys and girls (Mann-Whitney U test, *p* > 0.05).

Before further analysis, we will provide statistics on the distribution of children by approach in the formative experiment.

### Descriptive Statistics

The preschoolers’ EF level of development was taken into account when they were distributed according to the experimental conditions (see Table 1). Four experimental groups (“Traditional,” “Traditional with imaginary characters,” “Model-building,” “Symbolic”) and one control group were equalized in terms of the level of the children’s executive functions (Pearson’s Chi-square, *p* > 0.05). No statistically significant differences were found in the distribution of children based on EF level by approach. Children with different EF levels were divided proportionally into the experimental groups. In that way, further analysis of group differences is justified without taking into account the limitations in this part.

**Table 1:** Analysis of the distribution of students with different levels of EF into groups of the formative experiment

		Level of regulation			Total
		Low	Medium	High	
Control group	Quantity	5	11	6	22
	%	22.7%	50.0%	27.3%	100.0%
Symbolic approach	Quantity	5	14	6	25
	%	20.0%	56.0%	24.0%	100.0%
Model-building approach	Quantity	4	13	5	22
	%	18.2%	59.1%	22.7%	100.0%
Traditional approach	Quantity	5	13	5	23
	%	21.7%	56.5%	21.7%	100.0%
Traditional with imaginary characters	Quantity	6	13	5	24
	%	25.0%	54.2%	20.8%	100.0%
Total	Quantity	25	64	27	116
	%	21.6%	55.2%	23.3%	100.0%

*Pearson’s Chi-square = 0.718, p = 0.999*

Also, none of the identified groups differed in the pre-testing for mathematics (see Table 2). The lack of differences in the pre-testing is a prerequisite for correct interpretation of the results of the formative influence.

**Table 2:** Analysis of the distribution of students by mathematical abilities into groups in the formative experiment

	Control group	Symbolic approach	Model- building approach	Traditional approach	Traditional approach with imaginary characters	Kruskell-Wallis test
Final pre-test score	M+/–SD	M+/–SD	M+/–SD	M+/–SD	M+/–SD	*H* = 4,870;
	13.4+/–6.2	10.9+/–5.4	14.7+/–4.7	11.8+/–5.2	13.7+/–5.2	*P* = 0.300

After the formative part of the experiment, only children who had attended more than half of the sessions were included in the subsequent study. Eighty 80 children were tested in the post-test, and 44 in the delayed post-test. Although the number of children who participated on the post-test was lower than on the pre-test, the proportional distribution of EF levels within the experimental conditions was maintained (see Table 3). The groups remained equal according to this criterion, which removes further limitations on data analysis. There were 44 children in the delayed post-test: 12 children from the control group, 9 from the “Symbolic” approach, 11 from the “Model-building” approach, and 6 from the “Traditional” approaches.

**Table 3:** Analysis of the distribution of students with different levels of EF in the groups of the formative experiment based on the results of the post-test

		Level of regulation			Total post-test
		Low	Medium	High	
Control group	Quantity	5	9	5	19
	%	26.3%	47.4%	26.3%	100.0%
Symbolic approach	Quantity	3	10	5	18
	%	16%	55.6%	27.7%	100.0%
Model-building approach	Quantity	2	6	4	12
	%	16.7%	50.0%	33.3%	100.0%
Traditional approach	Quantity	3	5	4	12
	%	25.0%	41.7%	33.3%	100.0%
Traditional with imaginary characters	Quantity	5	9	5	19
	%	26.3%	47.4%	26.3%	100.0%
Total	Quantity	18	39	23	80
	%	21.5%	49.4%	29.1%	100.0%

*Pearson’s Chi-square = 0.209, p = 0.978*

### Analysis of the Effectiveness of Teaching Resources in the Formation of Concepts of Magnitudes

In order to assess the effectiveness of the formative sessions, we performed a nonparametric statistical analysis of the final scores of the pre- and post-tests for the experimental and control groups. The comparison showed significant differences in the total score for diagnostics of mathematical ability between the results of the pretest and post-test, and between the pre-test and delayed post-test (Wilcoxon Z test, p < 0.05). There were no significant differences found between the post-test and delayed post-test (Wilcoxon Z test, p > 0.05), which may suggest some stability in the results of the formative sessions (Wilcoxon Z test, p < 0.05). The minimum overall post-test score was in the control group — 2 points out of 31, with a minimum score of 7.5 for the experimental conditions. Thus, the results of both post-tests were significantly higher than the pre-test results.

We also note that the maximum score for diagnostics was indeed achieved on the post-test, with 29 points, and the maximum score for the delayed post-test, 27.5, demonstrates a slight decline. Since no significant differences were found between the post-test and delayed post-test, and the post-test had the larger variation in total score (2 to 29 for the post-test; 4.5 to 27.5 for the delayed post-test) and a larger sample, the post-test results will be considered for further analysis.

**Table 4:** Descriptive statistics of the final test score for the experimental and control groups.

		M	SD	Min	Max
Experimental groups	Pre-test	12.74	5.3	2	27
	Post-test	19.15	5	7.5	29
	Delayed post-test	18.04	6.47	4.5	27.50
Control group	Pre-test	13.4	6.2	2	25.5
	Post-test	15.6	7.3	2	29
	Delayed post-test	17.4	6.4	7	27

**Figure 1.**
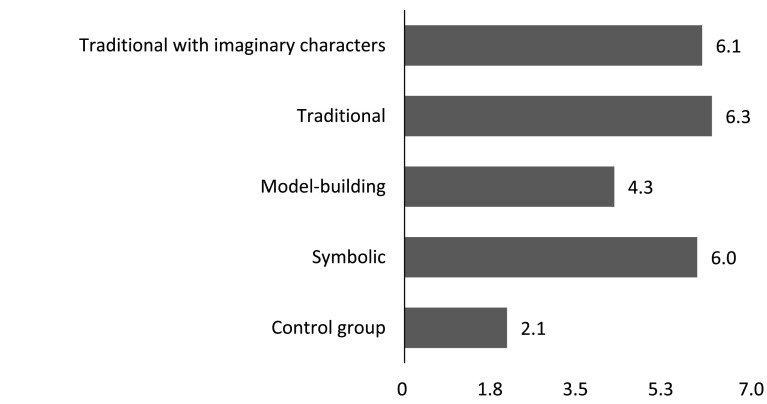
Increase in mean values for various approaches to formation.

*Note: Levene’s test, p > 0.05, ANOVA with non-parametric correction (robust criterion), p < 0.05*

To assess the effectiveness of specific teaching resources, we compared the increase in the final score on the post-test for each approach (see Table 5, Figure 1). We found that the type of formative lesson does indeed have a significant impact on the increase in the overall score for diagnosis of mathematical concepts and skills (ANOVA with non-parametric Welch correction, p < 0.05 with equality of variances, Levene’s criterion, *p* > 0.05).

**Table 5:** Analysis of multiple comparisons of approaches by increase in post-test score

(I) Approach	(J) Approach	Difference in mean increase values for (I–J)	Standard error	Significance, LSD
Control group	Symbolic	–5.8*	1.5	0.000
	Model–building	–2.2	1.7	0.2
	Traditional	–2.98	1.7	0.07
	Traditional imaginary characters with	–3.97*	1.5	0.010
Symbolic	Control	5.78*	1.5	0.000
	Model	3.6*	1.7	0.041
	Traditional	2.8	1.7	0.1
	Imaginary characters	1.8	1.5	0.2
Model-building	Control	2.2	1.7	0.2
	Symbol	–3.6*	1.7	0.041
	Traditional	–0.8	1.8	0.65
	Imaginary characters	–1.8	1.7	0.3
Traditional	Control	2.98	1.7	0.07
	Symbol	–2.8	1.7	0.1
	Model	0.8	1.8	0.65
	Imaginary characters	–0.98	1.6	0.5
Traditional with game elements	Control	3.97*	1.5	0.010
	Symbol	–1.8	1.5	0.2
	Model	1.8	1.7	0.3
	Traditional	0.98	1.6	0.5

*Note: Least significant difference (LSD) indicators of increase are in bold*.

Pairwise comparison of the increase in mean values for different approaches to formation showed that children who studied according to the “Symbolic” and “Traditional with imaginary characters” programs showed a significantly greater increase in total score on the post-test compared with the control group (LSD, *p* < 0.05). Significant differences were also found between children from the control group and children in the “Traditional” program (Mann-Whitney U-test, *p* < 0.05). The formative effect of the “Model-building” program does not show significant differences from the natural development of a child attending kindergarten (the control group) (see Table 5). We also note that children who attended formative sessions with the “Symbolic” approach showed a significantly greater increase in scores than those from the “Model-building” approach (LSD, *p* < 0.05).

For individual mathematical representations and actions, the following results were found:

1. The participants in the “Symbolic” approach showed a significantly greater increase in post-test scores than the children from the control group, in terms of the formation of concepts of length and area, the dependence of the number on the measurement; and the ability to select values and assemble “complex” sets (LSD, *p* < 0.05);
2. Children following the “Symbolic” approach also differed significantly in their formation of the concept of the dependence of the number on the measurement, from the children in the “Model-building” and “Traditional with imaginative characters” approaches (LSD, *p* < 0.05);
3. The increase in the total score for area and the ability to select magnitudes, in children who attended formative sessions according to the “Traditional with imaginative characters” program, was significantly greater than in children from the control group (LSD, *p* < 0.05);
4. There were no significant differences in the increase in the score on the post-test for volume, or the ability to use a measuring instrument to measure magnitudes (LSD, *p* > 0.05).

### Analysis of the Effectiveness of Teaching Resources Depending on the Level of EF

An analysis of variance with repeated measurements was performed to test the hypothesis that children with a lower level of EF will most effectively master the mathematical concepts of magnitudes when they are taught with the “Symbolic” approach. Table 6 shows the differential characteristics of differences between baseline test scores (pre-test) and subsequent ones (post-test). The joint interaction of the two factors — approach and level of EF — has a statistically significant effect on the increase in the total score on the post-test (ANOVA with nonparametric correction, *p* < 0.05).

**Table 6:** Differential characteristics of differences in test scores of final and baseline diagnostics for different approaches

	Low EF	Medium EF	High EF
Approach / statistic for increase of score	M+/–SD	M +/- SD	M +/- SD
Control group	–0.5+/–0.7	1.44+/–4.2	6+/–3.95
Symbolic	11.3+/–0.3	5.68+/–7.5	7.7+/–3.98
Model-building	4.75+/–6.7	1+/–2.2	9+/–1.78
Traditional	6.83+/–8.95	8+/–5.1	3.75+/–1.3
Traditional characters with imaginary	3.4+/–3.96	6.3+/–2.8	8.5+/–5.8
Total score			

ANOVA with nonparametric correction, F = 2.15, p = 0.04 for approach*EF level

Looking at the assessment of the mean values of differential differences, it is important to note that the greatest increase in scores among students with low EF was found in the formation of concepts of magnitude in the symbolic approach. The mean increase in the score of children with low EF in the symbolic approach was more than 11 points, with an overall average increase of 3.94, and this was the highest compared to other groups. We emphasize that children with low EF who did not attend formative sessions showed a tendency toward some decrease in scores on the post-test compared to the pre-test and showed the smallest increase compared to the other groups.

**Figure 2.**
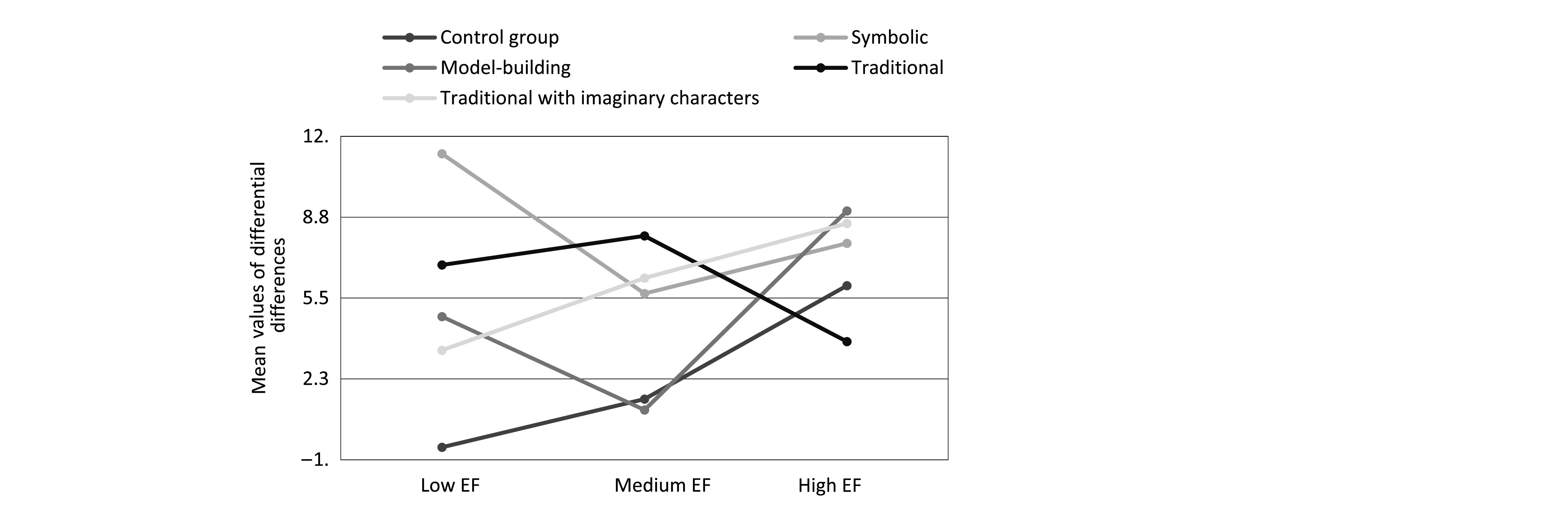
Profile plots for estimating the mean values of differential differences

To demonstrate the differences more clearly, Figure 2 shows profile plots for estimating the mean values of the differential differences.

An a posteriori analysis of the combined influence of the children’s executive functions and type of formative activities did not show statistically significant differences between children with the same level of EF who studied under the different programs.

However, children with a low level of self-regulation (executive function) mastered mathematical representations when following the “symbolic” approach significantly better than children from the control group and the “traditional with imaginary characters” approach (Welch’s t-test, *p* = 0.001 and *p* = 0.01, respectively). Preschoolers with a medium level of EFs mastered the material better when taught using both traditional programs than did the children from the control group and those taught according to the model-building approach (Welch’s t-test, *p* < 0.05 for each pair), and children with a medium level of self-regulation who studied with the “symbolic” program showed a significantly greater increase in scores than the control group (Welch’s t-test, *p* = 0.015). Participants with a high level of EF mastered the mathematical content in the Model-building approach more successfully than did the children from the Traditional with Imaginary Characters approach (Welch’s t-test, *p* = 0.004).

That said, we emphasize that, in general, the children with a high level of regulation showed a significantly greater improvement in mastery of mathematical concepts than those with a medium or low level (LSD, *p* < 0.05). Even in the control group, the scores of these children increased by an average of 6 points. On the other hand, children with low and medium levels of EF from the control group showed the least increase and even a worsening of results on the post-test. Pupils with a medium level of development of self-regulation who were taught with the “model-building” approach also showed an increase by an average of 1 point; that is, the level of formation of their concepts of magnitudes did not actually change.

## Discussion

An important result of this study was the significant increase in scores on the post-test compared to the pre-test, which indicates the effectiveness of our sessions. We have also seen that this effect has some stability, since a month after the experiment, the children completed the test just as successfully as they had immediately after the completion of the formative sessions. Besides the general developmental effect of the formative sessions, we also obtained significant differences among the approaches, and, accordingly, among the teaching resources used in them. The use of symbols and exemplars to form concepts of magnitude turned out to be the most productive and successful, whereas learning based on models did not differ in its impact from children’s natural development and what is learned from standard kindergarten classes. We attribute this result to the fact that operating with abstract signs of objects requires children to have quite developed visual-effective thinking and an internal plan of action (Venger, 1995). Therefore, for most children, this program may lie outside the zone of their actual and proximal development, since research has shown that the formation of abstract thinking only begins at the end of the preschool years (El’konin, 1989; Karabanova, 2005). We also emphasize that only the children who had been taught according to the “symbolic” approach coped with the making sets task (this action was not specially formed) significantly better than the control group, which once again indicates the effectiveness of the symbol as a teaching tool in expanding the child’s ZPD. Thus, our first hypothesis about the different effectiveness of the approaches was partially confirmed.

It is interesting that we did not find any increase in scores that differed from natural development in our testing for the concept of volume, the ability to use a measuring instrument, and to measure magnitudes. That is, formative sessions did not help children to master these actions specifically for a magnitude such as volume. Why is that? Volume is the most complex of the formed magnitudes. It is known that understanding of the conservation of volume arises much later than the conservation of length and quantity (Piaget, 1994). The formation of this concept is more difficult and occurs more slowly in preschoolers than the concepts of length and area (Obu khova, 1972), and some studies have shown that this concept does not lie within the ZPD of a preschooler (Veraksa et al., 2020). For volume as a magnitude, the strongest visual attributes (for example, the height of water in a jar, the width of a flask, etc.), prevent one from “grasping” the essential characteristics and fully mastering the concept. However, this result may be associated with incorrectly selected guidelines and actions for this concept in the programs and with methodological inaccuracies.

From analyzing the results, it can be asserted that the initial level of development of cognitive processes in older preschoolers is directly related to the children’s ability to learn new mathematical concepts. Children with a high level of EF are more successful in mastering new mathematical skills and concepts than those with a low and medium level of development of executive functions. This is consistent with earlier research findings that the level of EF is a predictor of the development of mathematical skills (Bull & Scerif, 2001; Clements et al., 2016; Jarvis, 2003; Veraksa et al., 2020).

The fact that children with a low level of EF scored the highest increase in points after completing their instruction according to the “symbolic” approach, and children with a high level of EF according to the “modeling” approach, may indicate the importance of choosing suitable tools for forming mathematical concepts, considering the level of formation of cognitive processes for student development (Clements et al., 2016; Veraksa et al., 2020) and confirms our second and third hypotheses.

It should also be emphasized that preschoolers with low EF who did not attend any special sessions showed worse results on the second test, which suggests the importance of special work with this group of children.

## Conclusion

We see the possibility of effective teaching even of children with a low level of voluntary self-regulation, by using a symbolic approach, whereby new ideas and concepts are introduced in a special emotionally constructed form, which makes it possible to motivationally include the children in learning and simplify their perception of the task (Veraksa et al., 2014). This approach is based on the use of symbols as a special form of mental representation of an object. Children use symbols in play as a means of self-expression (Veraksa et al, 2016). Mathematical instruction can also be symbolized to increase the effectiveness of the learning of children with low EF, whereas for children with high levels of cognitive development, more actions can be included in their mathematical instruction to operate with visual models, schematized representations of objects to stimulate their cognitive development (Venger, 1995).

## Limitations

Finally, we note that the limitations of this study include the experimenter effect, which could affect the results of the formative sessions and the entire study, as well as the relatively small number of children in each of the subgroups at the time of the post-test.

## References

[ref1] Aleksandrova, E.I. (2013). Psikhologo-pedagogicheskie osnovy postroeniia sovremennogo kursa matematiki [Psychological and pedagogical foundations of the modern mathematics education]. Nachal’naia shkola [Primary school], 1, 56–58.

[ref2] Aslanova, M.S., Buhalenkova, D.A., Veraksa, A.N., Gavrilova, M.N., Liucko, L.N., & Suhih, V.L. (2020). Traditsii i innovatsii v matematicheskom obrazovanii doshkol’nikov v Rossii: Sootvetstvuiut li oni obrazovatel’nym kriteriiam? [Traditions and innovations in mathematical education of preschoolers in Russia: do they meet the educational criteria?]. Vestnik Moskovskogo universiteta. Seriia 14. Psikhologiia [Moscow University Psychology Bulletin, Series 14. Psychology], 3, 166–193. 10.11621/vsp.2020.03.08

[ref3] Best, J.R., Miller, P.H., & Naglieri, J.A. (2011). Relations between executive function and academic achievement from ages 5 to 17 in a large, representative national sample. Learning and Individual Differences, 21(4), 327–336. 10.1016/j.lindif.2011.01.007 21845021 PMC3155246

[ref4] Bull, R., & Johnston, R.S. (1997). Children’s arithmetical difficulties: Contributions from processing speed, item identification, and short-term memory. Journal of Experimental Child Psychology, 65(1), 1–24. 10.1006/jecp.1996.2358 9126630

[ref5] Bull, R., & Scerif, G. (2001). Executive functioning as a predictor of children’s mathematics ability: Inhibition, switching, and working memory. Developmental Neuropsychology, 19(3), 273–293. 10.1207/S15326942DN1903_3 11758669

[ref6] Clements, D.H., Sarama, J., & Germeroth, C. (2016). Learning executive function and early mathematics: Directions of causal relations. Early Childhood Research Quarterly, 36, 79–90. 10.1016/j.ecresq.2015.12.009

[ref7] Davydov, V.V. (1962). Analiz stroeniia scheta kak predposylka postroeniia programmy po arifmetike [Analysis of the structure of the counting as a prerequisite for building an arithmetic program]. In D.B. Elkonin & V.V. Davydov (Eds.), Voprosy psikhologii uchebnoi deiatel’nosti mladshikh shkol’nikov (pp. 50–184). APN RSFSR Publ.

[ref8] Davydov, V.V. (1996). Teoriia razvivaiushchego obucheniia [Theory of developmental education]. Intor.

[ref9] Davydov, V.V., & El’konin, D.B. (1966). Vozrastnye vozmozhnosti usvoeniia znanii [Age-related learning opportunities]. Prosveshchenie.

[ref10] Davydov, V.V., Gorbov, S.F., Mikulina, G.G., & Savel’eva, O.V. (1996). Osobennosti kursa matematiki v sisteme razvivaiushchego obucheniia D.B. El’konina—V.V. Davydova [Mathematics course features in the system of developmental education D.B. Elkonin- V.V.Davydov]. Psikhologicheskaia nauka i obrazovanie [Psychological science and education], 1(4).

[ref11] Diamond, A. (2012). Executive functions. Annu. Rev. Psychol., 64, 135–154. 10.1146/annurev-psych-113011-143750 23020641 PMC4084861

[ref12] Duncan, G., Dowsett, C., Claessens, A., Magnuson, K., Huston, A., Klebanov, P., & Japel, C. (2007). School readiness and later achievement. Developmental Psychology, 43(6), 1428–1446. 10.1037/0012-1649.43.6.1428 18020822

[ref13] Elkonin, D.B. (1963). O teorii nachal’nogo obucheniia [About the theory of primary education]. Narodnoe obrazovanie [Public education], 4.

[ref14] Elkonin, D.B. (1978). Psikhologiia igry [Psychology of play]. Pedagogika.

[ref15] Elkonin, D.B. (1989). Izbrannye psikhologicheskie trudy [Selected psychological works]. Pedagogika.

[ref16] Espy, K.A., McDiarmid, M. M., Cwik, M. F., Stalets, M. M., Hamby, A., & Senn, T E. (2004). The contribution of executive functions to emergent mathematic skills in preschool children. Developmental Neuropsychology, 26(1), 465–486. 10.1207/s15326942dn2601_6 15276905

[ref17] Frolova, T.A. (1963). Opyt vvedeniia bukvennoi simvoliki pri obuchenii matematike v I klasse [The experience of introducing letter symbols in teaching mathematics in the first grade]. In Povyshenie effektivnosti obucheniia v nachal’noi shkole [Increasing the effectiveness of primary school education]. APN RSFSR publ.

[ref18] Galperin, P.Ya. (1975). Razumnost’ deistvii i predmet nauki [Reasonableness of actions and the subject of science]. In Psikhologicheskie issledovaniia, posviashchennye 85-letiiu so dnia rozhdeniia D.N. Uznadze [Psychological research dedicated to the 85th anniversary of the birth of D.N. Uznadze].

[ref19] Galperin, P.Ia. (1976). Vvedenie v psikhologiiu [Introduction to psychology]. Knizhniy dom ‘Universitet’.

[ref20] Galperin, P.Ia., & Georgiev, L.S. (1960). K voprosu o formirovanii nachal’nykh matematicheskikh poniatii [On the question of the formation of initial mathematical concepts]. Doklady APN RSFSR [Reports of the APN Rsfsr], 31–66.

[ref21] Jarvis, H.L., & Gathercole, S.E. (2003). Verbal and non-verbal working memory and achievements on national curriculum tests at 11 and 14 years of age. Educational and Child Psychology, 20(3), 123–140.

[ref22] Jordan, N., Kaplan, D., Ramineni, C., & Locuniak, M. (2009). Early math matters: Kindergarten number competence and later mathematics outcomes. Developmental Psychology, 45(3), 850–867. 10.1037/a0014939 19413436 PMC2782699

[ref23] Karabanova, O.A. (2005). Vozrastnaia psikhologiia. Konspekt lektsii [Developmental psychology. Lecture notes]. Airis-press.

[ref24] Korkman, M., Kirk, U., & Kemp, S.L. (2007). NEPSY II. Administrative Manual. Psychological Corporation.

[ref25] Leont’ev, A.N. (2000). Lektsii po obshchei psikhologii [Lectures on general psychology]. Smysl.

[ref26] Miyake, A., Friedman, N.P., Emerson, M.J., Witzki, A.H., Howerter, A., & Wager, T. (2000). The unity and diversity of executive functions and their contributions to complex “frontal lobe” tasks: A latent variable analysis. Cognitive Psychology, 41, 49–100. 10.1006/cogp.1999.0734 10945922

[ref27] Morosanova, V.I., Bondarenko I.N., Fomina T.G., & Velichkovsky B.B. (2021). Executive functions and conscious self-regulation as predictors of native language learning success in Russian middle school children. Journal of Siberian Federal University. Humanities & Social Sciences, 14(9), 1342–1354. 10.17516/1997-1370-0824

[ref28] Obukhova, L.F. (1972). Etapy razvitiia detskogo myshleniia [Stages of development of children’s thinking]. MSU publ.

[ref29] Piaget, J. (1994). Izbrannye psikhologicheskie trudy [Selected psychological works]. Prosve shchenie.

[ref30] Raven, D., & Kort, D.Zh. (2002). Rukovodstvo k progressivnym matritsam Ravena i slovarnym shkalam [Guide to Raven’s progressive matrices and vocabulary scales]. Cogito-center

[ref31] Ribner, A.D. (2020). Executive function facilitates learning from math instruction in kindergarten: Evidence from the ECLS-K. Learning and Instruction, 65, 101251. 10.1016/j.learninstruc.2019.101251

[ref32] Ritchie, S., & Bates, T. (2013). Enduring links from childhood mathematics and reading achievement to adult socioeconomic status. Psychological Science, 24(7), 1301–1308. 10.1177/0956797612466268 23640065

[ref33] Salmina, N.G.(1988). Znak i simvol v obuchenii [ Sign and symbol in education]. MSU publ.

[ref34] Sarama, J., & Clements, D.(2009). Building blocks and cognitive building blocks: Playing to know the world mathematically. American Journal of Play, 1(3), 313–337.

[ref35] Shapovalenko, I.V. (2004). Vozrastnaia psikhologiia [Developmental psychology]. Moscow: Gardariki.

[ref36] Shinelis, V.A., & Sidneva, A.N. (2020). Formirovanie orientirov v soderzhanii osnovnykh uchebnykh predmetov u doshkol’nikov: vozmozhnosti postroeniia programmy podgotovki k shkole, osnovannoi na printsipakh razvivaiushchego obucheniia [Formation of reference points in the content of the main academic subjects in preschoolers: the possibility of building a school preparation program based on the principles of developmental education]. In Materialy Mezhdunarodnogo molodezhnogo nauchnogo foruma “LOMONOSOV-2020” [Materials of the International Youth Forum “LOMONOSOV-2020”]. MSU publ.

[ref37] Shiian, O.A., Belolutskaia, A.K., Le-Van, T.N., & Zadadaev, S.A. (2021). Kognitivnoe razvitie doshkol’nikov: vzaimosviaz’ normativnyh, preobrazuiushchikh i simvolicheskikh sposobnostei [Cognitive development of preschool children: the relationship of normative, transformative and symbolic abilities]. Sovremennoe doshkol’noe obrazovanie [Modern preschool education], 6(108), 14–25. 10.24412/1997-9657-2021-6108-14-25

[ref38] Sidneva, A.N., Plotnikova, V.A., Solovieva, Iu., & Liutsko, L.N. (2021). Psikhologicheskii analiz uslovii i sredstv formirovaniia elementarnykh matematicheskikh predstavlenii u doshkol’nikov [Psychological analysis of conditions and means of elementary mathematical representations formation in preschoolers]. Vestnik Sankt-Peterburgskogo universiteta. Psikhologiia [Bulletin of St. Petersburg University, Psychology], 11(4), 389–408. 10.21638/spbu16.2021.407

[ref39] Smirnova, E.O. (2019). Specific features of modern preschool childhood. National Psychological Journal, 2(12), 25–32. 10.11621/npj.2019.0207

[ref40] Solovieva, Iu., Baltazar Ramos, A.M., & Quintanar Rojas, L. (2021). Experience in pre-school education in Mexico: Following L.S. Vygotsky. New Ideas in Child and Educational Psychology, 1(1), 77–95. 10.11621/nicep.2021.0104

[ref41] Swanson, H. L., & Sachse-Lee, C. (2001). Mathematical problem solving and working memory in children with learning disabilities: Both executive and phonological processes are important. Journal of Experimental Child Psychology, 3, 294–321. 10.1006/jecp.2000.2587 11394931

[ref42] Tikhomirova, T., Malykh, A., Lysenkova, I., Malykh, S. (2021). Cross-cultural Analysis of Models of the Relationship between the Cognitive Abilities and Academic Achievement in Primary School Education. Psychology in Russia: State of the Art, 14(4), 94–110. 10.11621/pir.2021.0407 36733813 PMC9888055

[ref43] Venger, L.A. (1986). Razvitie poznavatel’nykh sposobnostei v protsesse doshkol’nogo vospitaniia [Development of cognitive abilities in the process of preschool education]. Pedagogika.

[ref44] Venger, L.A. (1995). Programma “Odarennyi rebenok” (Osnovnye polozheniia) [The Gifted Child Program (Main provisions)]. Novaia shkola.

[ref45] Venger, L.A., & Kholmovskaia, V.V. (1978). Diagnostika umstvennogo razvitiia doshkol’nikov [Diagnostics of preschoolers mental development]. Pedagogika.

[ref46] Veraksa, A., Aslanova, M., Bukhalenkova, D., Veraksa, N., & Liutsko, L. (2020). Assessing the effective-ness of differentiated teaching resources for teaching math to preschoolers with different levels of executive functions. Education Science, 10(7), 181. 10.3390/educsci10070181

[ref47] Veraksa, A.N., Gorovaia, A.E., & Kisel’, A.V. (2014). Vozmozhnosti ispol’zovaniia znakovykh i simvolicheskikh sredstv v obuchenii doshkol’nikov (na primere osvoeniia fenomena radugi) [The possibilities of using signs and symbolic means in teaching preschoolers (on the example of mastering the rainbow phenomenon)]. Psikhologicheskaia nauka i obrazovanie [Psychological science and Education], 1, 19–34. 10.17759/psyedu.2014060202

[ref48] Veraksa, A. & Veraksa, N. (2016). Symbolic representation in early years’ learning: The acquisition of complex notions. European Early Childhood Education, 24(5), 668–683. 10.1080/1350293X.2015.1035539

[ref49] Veraksa, A.N., Yakupova, V.A., & Martynenko, M.N. (2015). Simvolizatsiia v strukture sposobnostei detei doshkol’nogo i shkol’nogo vozrasta [Symbolization in the structure of abilities of preschool and school-age children]. Kul’turno-istoricheskaia psikhologiia [Cultural and historical psychology], 11(2), 48–56. 10.17759/chp.2015110205

[ref50] Vygotsky, L.S. (2004). Psikhologiia razvitiia rebenka [Psychology of child development]. Smysl, Eksmo.

[ref51] Watts, T., Duncan, G., Clements, D., & Sarama, J. (2018). What is the long-run impact of learning mathematics during preschool? Child Development, 89(2), 539–555. 10.1111/cdev.12713 28105650 PMC5519454

[ref52] Watts, T., Duncan, G., Siegler, R., & Davis-Kean, P. (2014). What’s past is prologue: Relations between early mathematics knowledge and high school achievement. Educational Researcher, 43(7), 352–360. 10.3102/0013189X14553660 26806961 PMC4719158

[ref53] Zaporozhets, A.V. (1986). Izbrannye psikhologicheskie trudy [Selected psychological works]. Vol. 1. Peda gogika

[ref54] Zelazo, P.D. (2006). The Dimensional Change Card Sort (DCCS): A method of assessing executive function in children. Nature Protocols, 1(1), 297–301. 10.1038/nprot.2006.46 17406248

